# A short-term mindfulness and auricular acupressure program for preoperative sleep fragmentation in patients with spinal metastases: a retrospective quasi-experimental study

**DOI:** 10.3389/fpsyt.2026.1761276

**Published:** 2026-07-24

**Authors:** Dandan Li, Xiaomin Lang, Min Wang, Shaohua Liu, Kejie Ding, Xiaoqing Han, Hui Xu, Dengxing Lun, Yating Wen, Siying Li, Hongyu Wang

**Affiliations:** 1 Department of Spine Degeneration and Tumor, Weifang People’s Hospital, Weifang, China; 2 Department of Gastrointestinal Surgery, Weifang People’s Hospital, Weifang, China; 3 Department of Obstetrics, Weifang Maternal and Child Health Hospital, Weifang, China; 4 Department of Nursing, Weifang People’s Hospital, Weifang, China

**Keywords:** auricular acupressure, mindfulness-based stress reduction, non-pharmacological intervention, preoperative sleep disturbance, spinal metastases

## Abstract

**Objective:**

To evaluate the association of a short-term mindfulness-based stress reduction program combined with auricular acupressure with preoperative sleep fragmentation, anxiety, pain, and physiological stress responses in patients with spinal metastases using a retrospective quasi-experimental before-and-after design.

**Methods:**

This retrospective quasi-experimental before-and-after cohort study included patients with spinal metastases who underwent surgical treatment at Weifang People’s Hospital between January 2023 and January 2025. Patients admitted before implementation of the program were assigned to the control cohort, whereas patients admitted after program implementation were assigned to the intervention cohort. 92 patients were assigned to either the intervention group (n = 46), who received mindfulness-based stress reduction and auricular acupressure, or the control group (n = 46), who received standard care only. The intervention group received a one-week preoperative program combining short-term MBSR and auricular acupressure, delivered in two phases: outpatient instruction and home-based self-practice. A WeChat-based mini-program was used to deliver standardized mindfulness audio guidance and provide routine reminders during the intervention period. Auricular acupressure points were selected in accordance with national standards. The control group received standard perioperative care and educational materials, without any psychological or auricular interventions. Both groups were managed under the same pharmacological protocol. Outcome measures included subjective sleep quality (Athens Insomnia Scale, AIS), anxiety (Self-Rating Anxiety Scale, SAS), pain (Visual Analogue Scale, VAS), salivary cortisol levels, and objective sleep parameters—wake after sleep onset (WASO) and the number of nighttime awakenings—measured via a wearable device. Data were collected at baseline and prespecified time points before surgery.

**Results:**

The intervention group showed more favorable changes across several domains compared to the control group. Anxiety levels were significantly lower (SAS: 52.28 ± 2.75 vs. 56.20 ± 3.06, *P* < 0.001), as were pain scores (VAS: 4.20 ± 0.86 vs. 6.09 ± 1.35 preoperatively; 4.02 ± 0.88 vs. 5.52 ± 1.07 on admission; both *P* < 0.001) and salivary cortisol concentrations (580.24 ± 10.67 ng/dL vs. 609.43 ± 22.56 ng/dL, *P* < 0.001). Objective sleep indicators also favored the intervention, with reduced average WASO (30.00 ± 1.87 min vs. 39.87 ± 1.77 min, *P* < 0.001) and fewer nighttime awakenings (2.54 ± 0.21 vs. 3.55 ± 0.22, *P* < 0.001). These findings suggest that short-term MBSR combined with auricular acupressure may help improve sleep continuity indicators, reduce anxiety and pain, and modulate physiological stress responses in this patient population.

**Conclusion:**

The intervention was associated with improvements in anxiety, pain, salivary cortisol, and objective sleep continuity indicators, including reduced WASO and fewer nighttime awakenings. Although AIS scores were lower in the intervention group at an intermediate preoperative time point, the association between the intervention and AIS score change was attenuated after adjustment for covariates. Therefore, subjective insomnia outcomes should be interpreted cautiously.

## 1 Introduction

Patients with spinal metastases often experience substantial physiological and psychological stress due to tumor invasion, nerve compression, and persistent pain. Among various modifiable risk factors, preoperative fragmented sleep has gained increasing attention for its detrimental impact on surgical outcomes (1, 2). Studies indicate that approximately 65%–80% of these patients suffer from impaired sleep maintenance, characterized by frequent nocturnal awakenings and reduced sleep efficiency (3, 4). These disturbances not only exacerbate preoperative anxiety and pain sensitivity but also elevate the risk of postoperative complications, including delirium and infection. Despite the high prevalence of sleep disorders, perioperative management remains largely pharmacological, often relying on sedatives associated with adverse effects such as sedation, respiratory depression, and drug dependence—making them suboptimal choices for oncology patients. This underscores the urgent need for safe and effective non-pharmacological strategies to improve sleep quality in the perioperative period (5, 6). Fragmented sleep—defined as recurrent interruptions that prevent sustained restorative rest—is particularly common among patients with advanced cancer (7, 8). In those with spinal metastases, physical discomfort, psychological distress, and tumor-related symptoms contribute significantly to sleep disruption. Such fragmentation has been shown to impair immune function, heighten pain perception, and delay postoperative recovery. Emerging evidence suggests that the consequences of sleep disturbances in this population extend beyond the nighttime, affecting long-term emotional well-being, immunologic competence, and overall quality of life. Therefore, addressing fragmented sleep is critical to improving clinical outcomes and enhancing recovery in this vulnerable group.

Mindfulness-Based Stress Reduction (MBSR), a structured program that incorporates mindfulness meditation and attentional training, has been widely used in oncology to provide psychological support and relieve pain (9). Prior studies have shown that MBSR can significantly reduce anxiety and depressive symptoms while improving sleep quality in cancer patients (10). However, most of this research has focused on general psychological benefits, with limited exploration of MBSR as a targeted intervention for preoperative sleep issues in patients with spinal metastases. Additionally, the conventional 8-week MBSR protocol may be impractical for patients awaiting imminent surgery, highlighting the need for a shorter, more adaptable version (11–15). Auricular acupressure, a non-invasive technique based on traditional Chinese medicine, presents a simple, low-risk, and patient-friendly complementary approach (16–18).

In this study, we propose a combined intervention integrating short-term MBSR with auricular acupressure as a proactive strategy for managing preoperative sleep fragmentation. Delivered in multiple phases—outpatient prehabilitation, home-based practice, and inpatient reinforcement—this intervention aligns with the modern concept of perioperative “prehabilitation.” We hypothesize that this integrative approach offers a novel, non-pharmacological solution to enhance sleep quality and alleviate anxiety prior to surgery.

## 2 Materials and methods

### 2.1 Study design and participants

This study was designed as a non-randomized, retrospective quasi-experimental before-and-after cohort study conducted between January 2023 and January 2025. Patients admitted before the implementation of the short-term mindfulness-based stress reduction combined with auricular acupressure program were assigned to the control cohort, whereas patients admitted after the program was introduced were assigned to the intervention cohort. A total of 98 patients were initially screened, including 49 patients in the intervention period and 49 patients in the control period. Four patients in the intervention cohort were excluded because essential intervention process documentation required to confirm intervention exposure was incomplete, and two patients in the control cohort were excluded because they were transferred to other medical facilities before complete outcome data could be obtained. Finally, 92 patients were included in the statistical analysis, with 46 patients in the intervention group and 46 patients in the control group.

The inclusion criteria were as follows: (a) diagnosis of spinal metastases confirmed by pathological or imaging findings; (b) age between 18 and 80 years; (c) a preoperative waiting period of at least 7 days; (d) a score of ≥6 on the Athens Insomnia Scale (AIS) (19); (e) an estimated life expectancy of ≥3 months; and (f) availability of complete medical records including written informed consent. Exclusion criteria included: (a) use of sedative-hypnotics or antidepressants within 7 days prior to enrollment; (b) diagnosis of severe psychiatric disorders (e.g., schizophrenia or bipolar disorder); (c) dermatological conditions, infections, or allergic reactions involving the auricular region that could interfere with acupressure procedures; and (d) severe cardiopulmonary dysfunction or hearing impairment. Participants were excluded from the final analysis if essential intervention process documentation or key outcome data were incomplete, or if the surgical plan was changed during the preoperative period.

**Figure 1:**
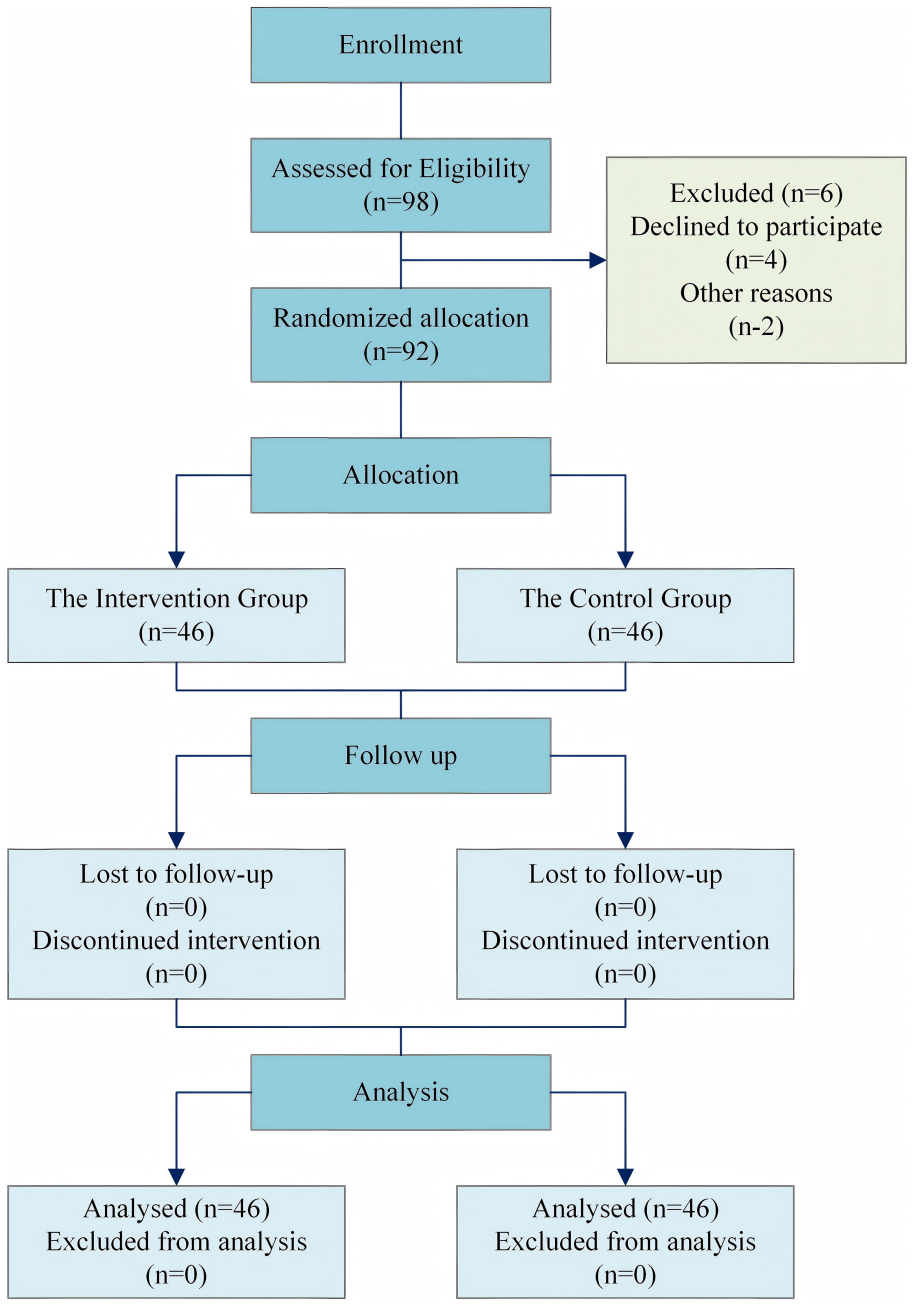
Participant flow diagram showing patient screening, exclusion, allocation, follow-up, and final analysis.

The timeline for enrollment, intervention, and assessment procedures is illustrated in **Figure 1**. **Table 1** presents a comparison of general patient information for both groups. Details of the sample size calculation and the formula used are provided in the **Supplementary Materials**. To reduce potential selection and temporal bias, the same inclusion and exclusion criteria were applied to both cohorts. All patients were recruited from the same department of the same hospital, and both groups were managed by the same multidisciplinary perioperative care team. The standard pharmacological protocol, perioperative nursing procedures, surgical indications, and routine patient education materials remained broadly consistent across the two study periods. All outcome data were retrospectively extracted from routinely collected clinical records, nursing assessment forms, wearable sleep-monitoring records, laboratory records, and available intervention process records. No prospective randomization was performed.

**Table 1:** Baseline and Outcome Comparisons Between Control and Intervention Groups.

Variable	All	Control Group	Intervention Group	P
Continuous Variables				
Age	56.77 ± 12.66	58.09 ± 13.51	55.46 ± 11.74	0.322
BMI	24.28 ± 2.53	24.11 ± 2.88	24.45 ± 2.15	0.529
SAS Before Intervention	55.51 ± 3.21	55.28 ± 3.45	55.74 ± 2.97	0.498
SAS on Admission Day	54.24 ± 3.50	56.20 ± 3.06	52.28 ± 2.75	<0.001
AIS Before Intervention	6.52 ± 1.19	6.39 ± 1.20	6.65 ± 1.18	0.296
AIS 3 Days Before Surgery	6.74 ± 1.24	7.13 ± 1.17	6.35 ± 1.20	0.002
AIS on Admission Day	7.07 ± 1.16	7.26 ± 1.36	6.87 ± 0.88	0.105
VAS Before Intervention	5.53 ± 0.91	5.67 ± 1.13	5.39 ± 0.58	0.137
VAS 3 Days Before Surgery	5.14 ± 1.47	6.09 ± 1.35	4.20 ± 0.86	<0.001
VAS on Admission Day	4.77 ± 1.23	5.52 ± 1.07	4.02 ± 0.88	<0.001
SC Level Before Intervention	601.34 ± 11.53	600.35 ± 11.60	602.33 ± 11.50	0.414
SC Level on Admission Day	594.84 ± 22.88	609.43 ± 22.56	580.24 ± 10.67	<0.001
Mean WASO	34.94 ± 5.28	39.87 ± 1.77	30.00 ± 1.87	<0.001
Mean Number of Awakenings	3.05 ± 0.55	3.55 ±0.22	2.54 ±0.21	<0.001
Categorical variables				
Gender				0.676
Male	53.3% (49)	50.0% (23)	50.0% (23)	
Female	46.7% (43)	43.5% (20)	56.5% (26)	
Education Level				0.960
Primary school or below	8.7% (8)	8.7% (4)	8.7% (4)	
Junior high or vocational school	75% (69)	73.9% (34)	76.1% (35)	
High school or above	16.3% (15)	17.4% (8)	15.2% (7)	
Primary Caregiver				0.517
Parents	16.3% (15)	15.2% (7)	17.4% (8)	
Siblings	25% (23)	21.7% (10)	28.3% (13)	
Spouse	53.3% (49)	54.3% (25)	52.2% (24)	
Children	5.4% (5)	8.7% (4)	2.2% (1)	
History of Chemotherapy/Radiotherapy				1
Yes	42.4% (39)	41.3% (19)	43.5% (20)	
No	57.6% (53)	58.7% (27)	56.5% (26)	
Residence				0.970
Urban	35.9% (33)	37.0% (17)	34.8% (16)	
County	28.3% (26)	28.3% (13)	28.3% (13)	
Rural	35.9% (33)	34.8% (16)	37.0% (17)	
Marital Status				0.261
Unmarried	5.4% (5)	6.5% (3)	4.3% (2)	
Married	82.6% (76)	87.0% (40)	78.3% (36)	
Widowed	12% (11)	6.5% (3)	17.4% (8)	

### 2.2 Ethical approval

This study involved non-pharmacological behavioral interventions and was considered to pose minimal risk to participants. The study protocol was reviewed and approved by the Ethics Committee of Weifang People’s Hospital (Approval No. KYLL20250826-6). The study was conducted in accordance with the ethical standards of the institutional research committee and the principles outlined in the 1964 Declaration of Helsinki and its later amendments. Strict measures were implemented to protect participant confidentiality, and all data were anonymized to ensure privacy. Any modifications to the study protocol—including changes in procedures, outcome assessments, interventions, or statistical methods—were required to be formally submitted in writing and approved by the Institutional Review Board.

### 2.3 The intervention group

Patients in the intervention group received a combined short-term Mindfulness-Based Stress Reduction (MBSR) and auricular acupressure program, implemented in two phases: centralized outpatient training followed by a 7-day home-based self-practice period. The intervention followed a “compressed-time and function-focused” approach to maximize feasibility and adherence. During the outpatient phase, a certified mindfulness therapist provided on-site instruction for the first mindfulness-based stress reduction (MBSR) practice, which lasted approximately 40 minutes and included 20 minutes of face-to-face guidance followed by 20 minutes of self-practice. The initial exercise focused on the “breath anchoring” technique, aimed at helping patients concentrate on their breathing and acquire relaxation skills.

**Figure 2:**
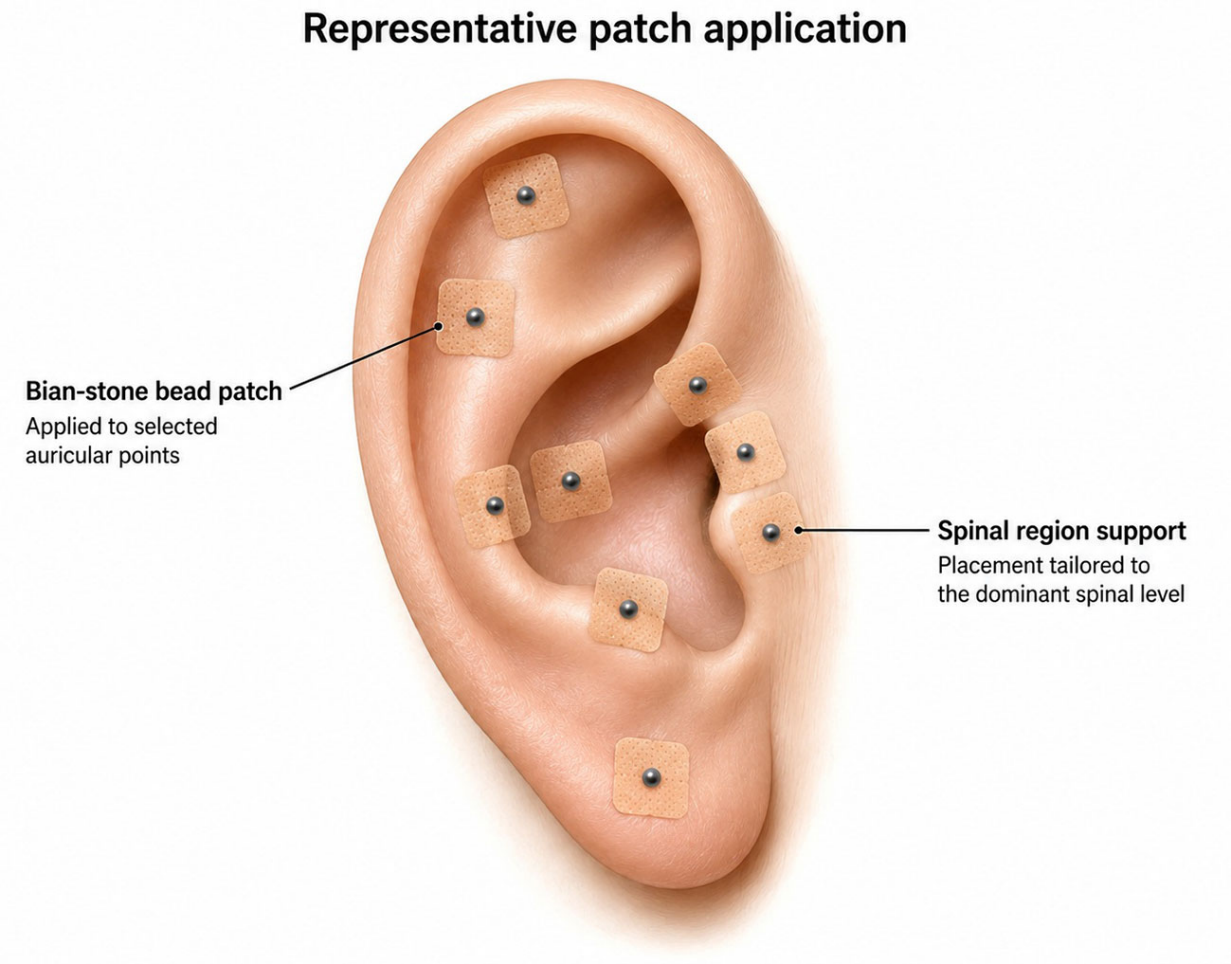
Representative auricular acupressure patch application. Bian-stone bead patches were applied to selected auricular points, including an individualized vertebral-related auricular region corresponding to the dominant spinal level.

**Figure 3:**
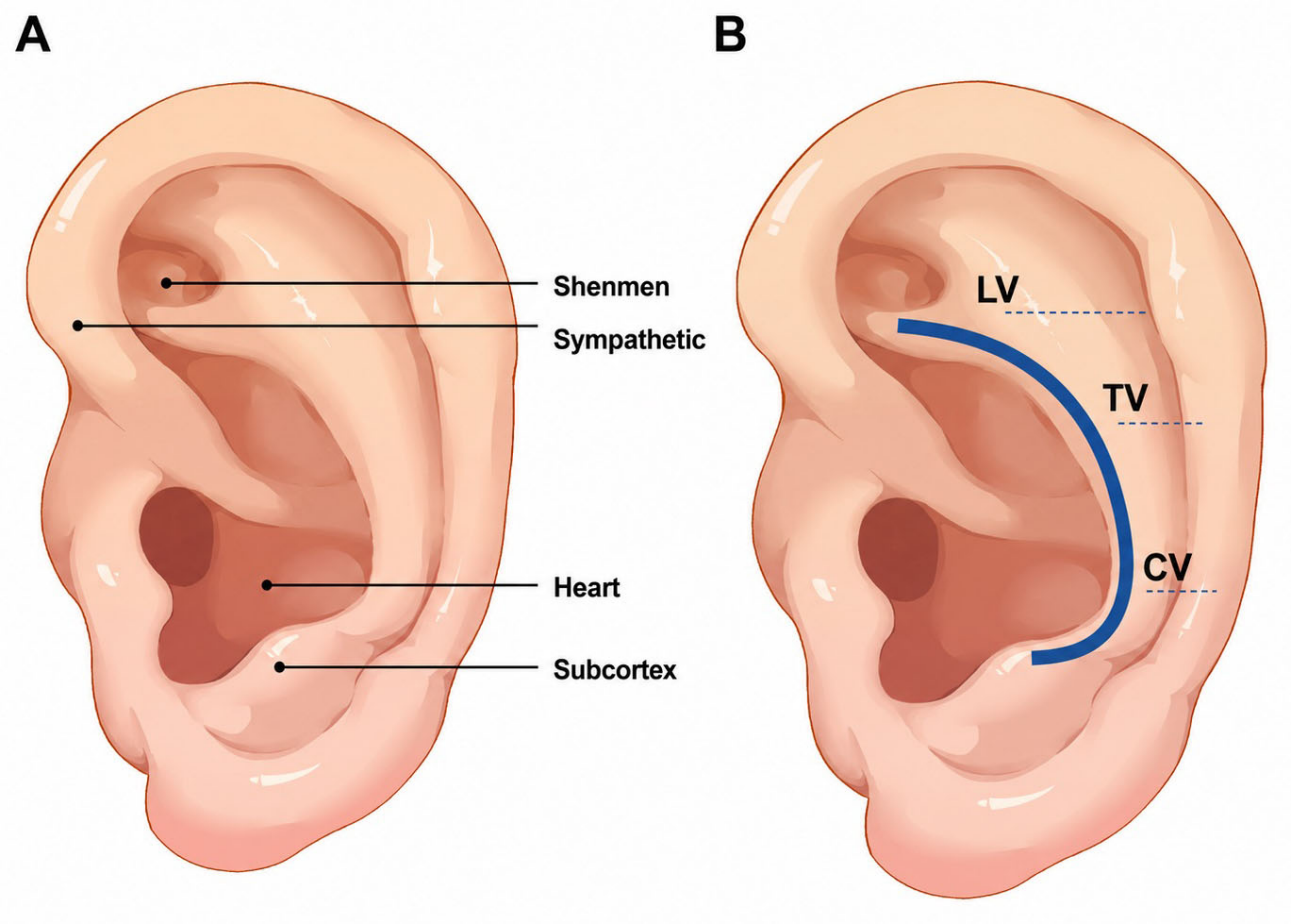
Auricular point localization used in the study. **(A)** Core regulatory auricular points used in the intervention, including Shenmen, Sympathetic, Heart, and Subcortex. **(B)** Schematic representation of the vertebral-related auricular regions used for individualized point selection. LV, lumbar vertebrae; TV, thoracic vertebrae; CV, cervical vertebrae.

The researcher explained the underlying principles and mechanism of auricular acupressure, as well as the rationale for selecting specific auricular points. Patients were instructed on how to reposition or replace the acupressure patches each morning and were taught the corresponding self-care steps. A live demonstration was conducted on the concentric-circle disinfection technique using 75% alcohol, after which patients were guided to perform auricular acupressure patch application independently to ensure they had mastered the procedure. At the end of the session, the research nurse distributed a standardized home-practice manual and an audio guide recorded by the mindfulness therapist. Each patient also received an auricular acupressure toolkit, which included a point location card, marker pen, locator stick, medical-grade acupoint pressure stimulation patches containing Bian-stone beads, and alcohol swabs (see **Figures 2**, **3**).

Auricular point selection was based on national nomenclature and location standards for auricular points, traditional auricular therapy principles, and auricular somatotopic mapping principles originally described by Paul Nogier, in which the auricle is viewed as a microsystem representing the body in an inverted fetal configuration (20). Core regulatory points included Shenmen, Subcortex, Sympathetic, and Heart. Shenmen was selected because it is commonly used in auricular therapy for calming, anxiolytic, analgesic, and sleep-regulating purposes. Subcortex refers to a standardized auricular point used in Chinese auricular acupuncture systems; this term does not imply direct anatomical stimulation of the cerebral subcortex, but denotes an auricular regulatory point traditionally used for sleep disturbance, pain, and autonomic-emotional dysregulation. The Sympathetic point was selected to support autonomic balance, and the Heart point was selected according to traditional auricular therapy principles related to sleep and emotional regulation.

Spinal somatotopic points were selected according to the anatomical level of spinal involvement. The cervical, thoracic, lumbar, and sacral vertebral correspondences were represented along the antihelix, and the point corresponding to the dominant symptomatic or surgically targeted spinal level was selected. When multiple spinal levels were involved, the clinically dominant level was prioritized.

Participants were instructed to apply gentle pressure to each selected point for approximately 30 seconds, three times daily. This schedule was selected as a pragmatic, low-burden perioperative stimulation protocol intended to provide repeated but tolerable stimulation while minimizing discomfort, fatigue, and excessive manipulation of the auricle in patients with advanced cancer. Previous auricular acupressure studies have used heterogeneous stimulation schedules, commonly ranging from once to several times daily and from approximately 30 seconds to several minutes per stimulation episode. The product was a single-bead patch formulation, 600 patches per package, model I-1, manufactured by Hengshui Huayun Medical Device Co., Ltd. (Hengshui, China).

In the subsequent home-based phase, participants were instructed to perform daily mindfulness exercises and auricular acupressure patch pressing in parallel. Compliance and remote supervision were supported via a WeChat mini-program and a group messaging platform. Detailed daily themes and tasks are outlined in **Table 2**. To accommodate pain-related limitations, mindfulness sessions were adapted to allow patients to choose postures that minimized discomfort. To further promote adherence, a dedicated module within the WeChat mini-program was developed to deliver daily reminders and track progress. Upon enrollment, each participant was instructed to install the app and register using their study ID. From Day –7 to Day –1 before surgery, the system automatically pushed daily notifications at 8:00 AM, summarizing the practice objectives and linking directly to the day’s audio session. If a participant did not mark the task as completed within the specified time, a follow-up reminder was sent after one hour, and the research nurse was alerted for additional contact. The WeChat mini-program was used primarily to support intervention delivery, reminders, and routine process monitoring during clinical practice. Although some process information was available during routine care, detailed structured adherence metrics, such as exact session completion counts, cumulative practice duration, and response time to reminders, were not uniformly archived for all participants. Participants who repeatedly failed to complete sessions received personalized follow-up by phone to encourage continued engagement.

**Table 2:** Daily home-based mindfulness and auricular acupressure intervention protocol.

Home-based intervention phase	Practice theme	Main content description	Mindfulness practice frequency and duration	Auricular acupressure procedure and frequency
Day 1	Breath Awareness	Focus all attention on the natural process of breathing, observing the rhythm, depth, and direction of each inhalation and exhalation. When attention drifts, gently guide it back to the breath to cultivate present-moment awareness and alleviate preoperative anxiety.	Once daily, ~30 minutes; audio-guided + self-practice	Each morning, self-locate and apply seeds to primary and secondary auricular points; press each point 3 times/day, 30 seconds each time
Days 2–3	Body Scan	Patients are guided to sequentially shift attention to different body regions, noticing sensations such as tension or pressure without judgment. This practice enhances awareness and tolerance of bodily experiences.	Once daily, ~30 minutes; audio-guided + self-practice	Same as above
Days 4–5	Pain Acceptance	Focus on the site of pain, accept the present sensation, and reduce anxiety related to pain.	Once daily, ~30 minutes; audio-guided + self-practice	Same as above
Days 6–7	Scenario Rehearsal	Simulate the preoperative process to regulate preoperative anxiety.	Once daily, ~30 minutes; audio-guided + self-practice	Same as above
Full Cycle	Mini Mindfulness Practices	Breath awareness, quick body scans, etc., to reinforce mindfulness skills.	At least twice daily, 1–5 minutes each	______________________
Full Cycle	Auricular Acupressure Compliance Management	Daily reapplication of auricular seeds at primary points (Shenmen, Subcortex, Heart, Sympathetic) and secondary points (lumbar/thoracic/cervical regions) each morning.	__________________	Each morning, reapply seeds; press each point 3 times/day, 30 seconds each time, until slight soreness is felt

### 2.4 The control group

Both the intervention and control groups received identical standard pharmacologic care, administered by the same team of anesthesiologists according to a unified protocol to ensure consistency and minimize intergroup variability in medication management. The groups differed only in the non-pharmacological component. Participants in the control group received routine perioperative education through a standardized informational booklet covering surgical procedures, pain management strategies, and postoperative rehabilitation. This booklet was identical to that provided to the intervention group, except that it excluded the psychological intervention content. All instructions and care procedures were delivered by the same nursing team, with equivalent frequency, duration, and presentation format. This design was intended to minimize potential placebo effects and operator-related bias.

### 2.5 Outcome measures

Outcome assessments included six domains covering baseline characteristics, objective sleep metrics, psychological symptoms, and biological stress markers. A combination of subjective and objective measures was employed to evaluate the effectiveness of the intervention:

#### 2.5.1 Demographic and clinical characteristics

Baseline information, including sex, age, marital status, education level, place of residence, and history of prior treatments, was collected using a structured questionnaire.

#### 2.5.2 Objective sleep parameters

All participants wore a Huawei Watch GT 3 smartwatch for seven consecutive nights prior to surgery. Sleep data were automatically recorded via the TruSleep™ system, focusing on two key indicators: wake after sleep onset (WASO) and the number of nighttime awakenings (21). Following the intervention, the research team extracted these data and calculated 7-day average values for each participant to assess sleep continuity and fragmentation. The Huawei Watch GT 3 TruSleep™ system was selected because it is non-invasive, easy to operate, suitable for repeated nightly monitoring, and feasible for perioperative oncology patients who may have pain, limited mobility, or difficulty tolerating laboratory-based polysomnography. In this study, wearable-derived sleep data were used as supportive objective indicators of sleep continuity and fragmentation rather than as diagnostic measures for sleep disorders.

#### 2.5.3 Athens insomnia scale

The AIS, a validated self-reported questionnaire, was used to assess insomnia symptoms. Scores were collected at three time points: baseline (prior to the intervention), three days before surgery, and one day before surgery (19, 22, 23).

#### 2.5.4 Self-rating anxiety scale

Anxiety levels during the previous week were assessed using the SAS. Higher scores reflect greater severity of anxiety symptoms (24, 25). Assessments were conducted at baseline and upon hospital admission.

#### 2.5.5 Visual analogue scale for pain

Pain intensity was evaluated using a 0–10 visual analogue scale (VAS), where 0 represented no pain and 10 the most severe pain imaginable (26, 27). Participants rated their pain at baseline, three days before surgery, and one day before surgery.

#### 2.5.6 Salivary cortisol

Salivary cortisol, a biomarker of physiological stress and sleep disturbance, was measured given its known association with impaired sleep quality and heightened stress reactivity (28). Samples were collected at baseline and on the day of hospital admission.

## 3 Statistical methods and results

### 3.1 Baseline characteristics of participants

A total of 92 patients were included in the final analysis, with 46 participants assigned to the intervention group and 46 to the control group. Prior to the intervention, there were no statistically significant differences between the two groups in demographic or clinical characteristics, indicating good baseline comparability (all *P* > 0.05).

The mean age was 55.46 ± 11.74 years in the intervention group and 58.09 ± 13.51 years in the control group (*P* = 0.322). The mean body mass index (BMI) was 24.45 ± 2.15 and 24.11 ± 2.88 in the intervention and control groups, respectively (*P* = 0.529). Female participants comprised approximately 53.3% of the overall cohort, with no significant difference in gender distribution between groups (*P* = 0.676). Similarly, no significant intergroup differences were observed in terms of education level (*P* = 0.960), primary caregiver (*P* = 0.517), history of prior radiotherapy or chemotherapy (P = 1.000), place of residence (P = 0.970), or marital status (*P* = 0.261). These results confirm the demographic and clinical equivalence of the two groups at baseline, thereby supporting the validity of subsequent comparisons of intervention effects (**Table 1**).

### 3.2 Comparison of anxiety, sleep quality, and pain perception between groups

Independent-samples t-tests were used to compare continuous outcome variables between the intervention and control groups. These included subjective sleep-related measures (AIS, SAS, and VAS for pain), physiological stress markers (SC), and objective sleep parameters—specifically, WASO and the average number of nighttime awakenings recorded over the 7-day period. Results are presented as mean ± standard deviation (SD), with statistical significance set at *P* < 0.05.

During the post-intervention period leading up to hospital admission, the intervention group showed more favorable changes than the control group across multiple domains. On the day of admission, the intervention group had a significantly lower mean SAS score (52.28 ± 2.75 vs. 56.20 ± 3.06, *P* < 0.001) and lower salivary cortisol levels (580.24 ± 10.67 ng/dL vs. 609.43 ± 22.56 ng/dL, *P* < 0.001). Subjective sleep quality, assessed by the AIS three days prior to surgery, was also significantly better in the intervention group (6.35 ± 1.20 vs. 7.13 ± 1.17, *P* = 0.002).

**Figure 4:**
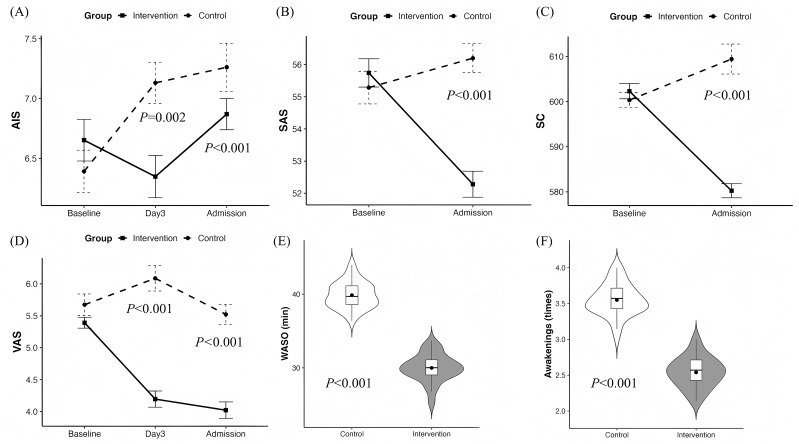
Changes in sleep quality, anxiety, physiological stress, pain, and objective sleep-continuity indicators between the intervention and control groups. **(A)** Athens Insomnia Scale scores at baseline, Day 3, and hospital admission. **(B)** Self-Rating Anxiety Scale scores at baseline and hospital admission. **(C)** Salivary cortisol levels at baseline and hospital admission. **(D)** Visual Analogue Scale pain scores at baseline, Day 3, and hospital admission. **(E)** Wake after sleep onset during the 7-day preoperative monitoring period. **(F)** Average nighttime awakenings during the 7-day preoperative monitoring period.

Pain perception, as measured by the VAS, was significantly reduced in the intervention group both three days before surgery (4.20 ± 0.86 vs. 6.09 ± 1.35, *P* < 0.001) and on the day of admission (4.02 ± 0.88 vs. 5.52 ± 1.07, *P* < 0.001). In terms of objective sleep parameters recorded on the night of admission, the intervention group showed significantly lower WASO (30.00 ± 1.87 min vs. 39.87 ± 1.77 min, *P* < 0.001) and fewer nighttime awakenings (2.54 ± 0.21 vs. 3.55 ± 0.22, *P* < 0.001) (**Figures 4A–F**).

### 3.3 Multiple linear regression analysis of intervention effects on sleep and related outcomes

Considering the relatively small sample size and the risk of model overfitting, covariate adjustment was restricted to clinically and demographically relevant variables that were consistently available in the retrospective records. Three models were constructed sequentially. Model 1 was unadjusted; Model 2 was adjusted for age and sex; and Model 3 was further adjusted for BMI, education level, primary caregiver, history of radiotherapy or chemotherapy, place of residence, and marital status. More detailed disease-related variables, such as tumor stage, tumor burden, anatomical severity of spinal metastasis, and exact opioid dosage, were not consistently available across all records and therefore could not be reliably incorporated into the regression models.

To further evaluate the impact of the intervention on sleep quality and related outcomes, multiple linear regression analyses were performed with group assignment (intervention = 1, control = 0) as the independent variable. The dependent variables included changes in AIS, SAS, VAS scores, and SC levels (each calculated as the difference between values at hospital admission and baseline), as well as the average WASO and the average number of nighttime awakenings during the 7-day intervention period.

Three models were constructed sequentially:

Model 1: unadjusted model;

Model 2: adjusted for age and sex;

Model 3: further adjusted for BMI, education level, primary caregiver, history of radiotherapy or chemotherapy, place of residence, and marital status.

The analyses demonstrated that the intervention group exhibited significant improvements across several emotional and sleep-related indicators compared to the control group. In the unadjusted model (Model 1), the intervention was associated with significantly lower SAS scores (β = –4.37, 95% CI: –6.36 to –2.37, *P* = 4.28×10^-5^), lower VAS scores (β = –1.21, 95% CI: –1.77 to –0.65, *P* = 4.92×10^-5^), and reduced salivary cortisol levels (β = –31.17, 95% CI: –39.61 to –22.73, *P* = 1.44×10^-^¹^0^). These associations remained robust after adjustment for covariates in Models 2 and 3.

The effect of the intervention on AIS scores showed a marginal trend toward significance in the unadjusted model (*P* = 0.07), which diminished after full adjustment (Model 3: β = –0.45, *P* = 0.20). In contrast, the intervention had a pronounced impact on objective sleep parameters: Model 3 revealed a significant reduction in average WASO (β = –9.68, *P* = 2.18×10^-40^) and the number of nighttime awakenings (β = –0.98, *P* = 1.97×10^-^³^5^), indicating a clear benefit in promoting sleep continuity (**Table 3**).

**Table 3:** Linear regression analysis of the relationship between mindfulness-based interventions and six outcome indicators.

Outcome indicator	Model 1	*P*	Model 2	*P*	Model 3	*P*
AIS	-0.65 (-1.34 ~ 0.04)	0.07	-0.58 (-1.27 ~ 0.10)	0.10	-0.45 (-1.17 ~ 0.26)	0.20
SAS	-4.37 (-6.36 ~ -2.37)	4.28E-05	-4.34 (-6.36 ~ -2.32)	5.96E-05	-3.92 (-6.00 ~ -1.83)	4.07E-04
VAS	-1.21 (-1.77 ~ -0.65)	4.92E-05	-1.18 (-1.74 ~ -0.62)	8.54E-05	-1.13 (-1.71 ~ -0.55)	2.64E-04
SC	-31.17 (-39.61 ~ -22.73)	1.44E-10	-31.67 (-40.20 ~ -23.13)	1.38E-10	-31.63 (-40.49 ~ -22.78)	6.26E-10
WASO	-9.86 (-10.61 ~ -9.12)	1.28E-43	-9.81 (-10.56 ~ -9.06)	1.41E-42	-9.68 (-10.43 ~ -8.93)	2.18E-40
Awakenings	-1.00 (-1.09 ~ -0.92)	1.99E-38	-1.01 (-1.10 ~ -0.92)	1.43E-37	-0.98 (-1.07 ~ -0.89)	1.97E-35

The data are presented as regression coefficients and their 95% confidence intervals (β, 95% CI). The P - values are expressed in scientific notation. Model 1 is the unadjusted model. Model 2 adjusts for age and gender. Model 3 further adjusts for covariates such as body mass index (BMI), educational attainment, primary caregiver, history of previous radiotherapy and chemotherapy, place of residence, and marital status based on Model 2.

### 3.4 Mediation analysis of the intervention’s effects on sleep improvement

To examine whether the intervention indirectly improved sleep through reductions in anxiety or pain, structural equation modeling was employed to assess mediation pathways. The intervention group was treated as the independent variable (X), with change in Athens Insomnia Scale score (AIS_change) as the primary outcome (Y). Changes in Self-Rating Anxiety Scale score (SAS_change) and Visual Analogue Scale score (VAS_change) were included as potential mediators (M).

Two mediation models were constructed:

Intervention → SAS_change → AIS_change.

Intervention → VAS_change → AIS_change.

All models were estimated using maximum likelihood methods with 1, 000 bootstrap samples to compute confidence intervals and assess the significance of indirect effects. Path coefficients (a, b, and c’) and corresponding p-values were reported to evaluate mediation effects.

In the model with SAS_change as the mediator, the intervention significantly reduced anxiety levels (a = –3.92, *p* < 0.001), and improvements in anxiety were significantly associated with improvements in AIS scores (b = 0.23, *p* = 0.041). The direct effect of the intervention on AIS_change was marginally significant (c’=–0.42, *p* = 0.081), suggesting that SAS_change partially mediated the intervention’s effect on sleep quality.

**Figure 5:**
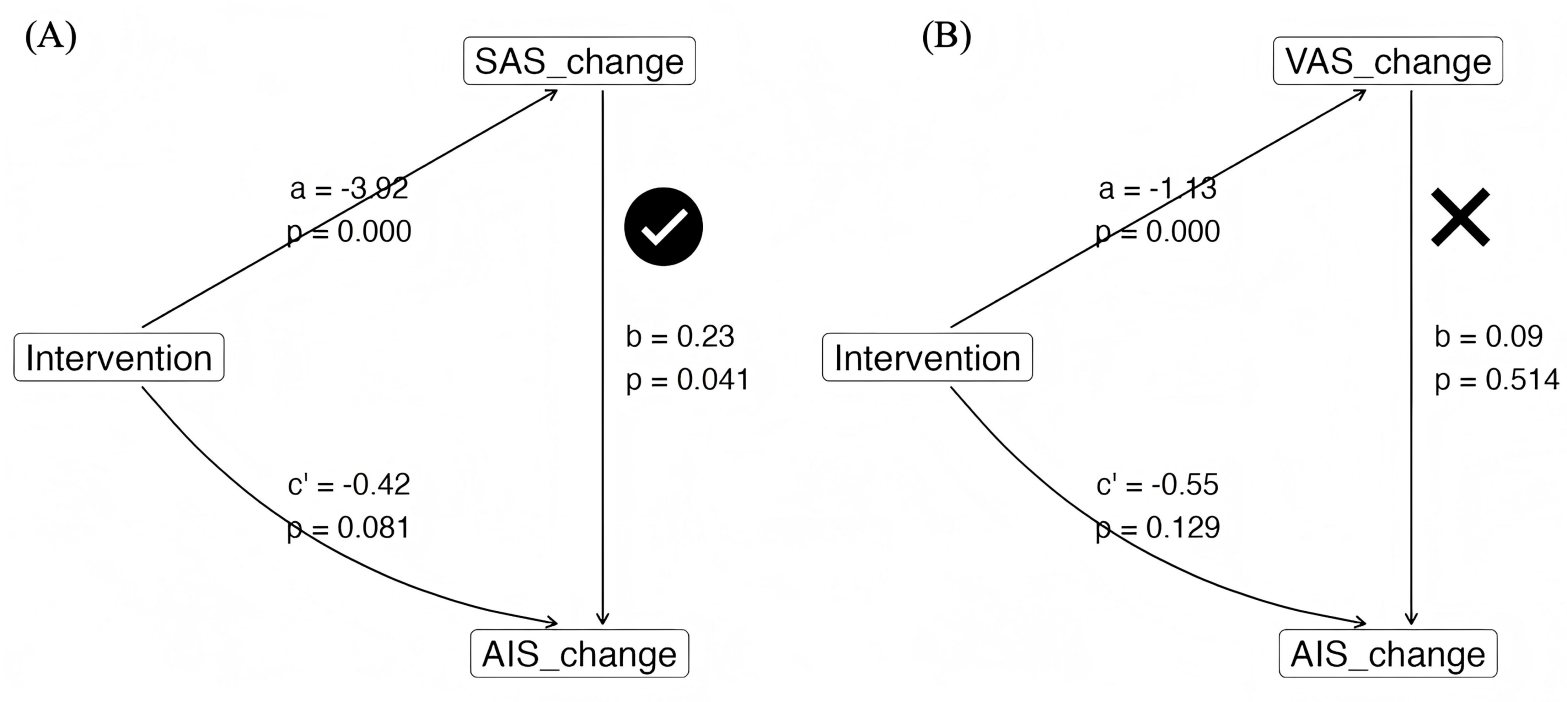
Mediation analysis of the intervention effect on AIS_change through SAS_change and VAS_change. **(A)** Mediation model with SAS_change as the mediator. **(B)** Mediation model with VAS_change as the mediator. Path coefficients and p-values are shown. The SAS_change pathway was statistically significant, whereas the VAS_change pathway was not statistically significant.

In contrast, while the intervention had a significant impact on VAS_change (a = –1.13, *p* < 0.001), the association between VAS_change and AIS_change was not statistically significant (b = 0.09, *p* = 0.514), indicating that pain reduction did not mediate the relationship between the intervention and sleep improvement (**Figure 5**).

## 4 Discussion

Previous studies have demonstrated that mindfulness-based interventions can effectively alleviate anxiety, improve subjective sleep quality, and enhance emotional regulation and coping capacity among cancer patients during the perioperative period (15, 29–32). The present study showed that the combined intervention was associated with significant improvements in objective sleep continuity indicators, including WASO and nighttime awakenings, as well as reductions in anxiety, pain, and salivary cortisol. Although AIS scores were lower in the intervention group at three days before surgery, the association between the intervention and AIS score change was attenuated after covariate adjustment. This suggests that the intervention may have a more robust effect on sleep continuity and stress-related physiological arousal than on subjective insomnia symptoms measured by AIS.

The intervention was delivered in three consecutive phases—outpatient, home-based, and inpatient—ensuring a continuum of psychological support. In the outpatient phase, patients received structured guidance in either one-on-one or group sessions, which helped them understand and begin practicing core mindfulness techniques. In particular, methods such as breath awareness and body scanning enabled patients to shift attention to present-moment sensations and reduce anticipatory anxiety surrounding surgery (2, 33). Given the common occurrence of emotional states such as fear, tension, and helplessness before surgery, early mindfulness training helped patients recognize and accept negative emotions, reducing preoperative stress reactivity (34, 35). The home-based phase served as an essential bridge, reinforcing the mindfulness routine in patients’ daily environments. Practicing at fixed times each day in a familiar setting not only facilitated the internalization of mindfulness techniques but also promoted autonomy and consistency in emotional regulation. Compared to facility-based interventions alone, home-based practice provided enhanced flexibility in posture and timing—particularly important for patients experiencing pain or fatigue—thus improving intervention adherence. The WeChat mini-program was used to provide automated reminders and access to daily audio sessions during routine perioperative nursing practice. For patients who required additional support, telephone follow-up was used to encourage continued participation. These process-support strategies may have helped maintain participant engagement and intervention continuity; however, structured adherence metrics were not uniformly archived, and the relationship between intervention exposure and outcomes could not be quantitatively evaluated in this retrospective analysis.

This hybrid delivery model combining digital reminders with personalized supervision may help support intervention engagement in routine perioperative nursing practice (32, 36). Mindfulness practices performed prior to bedtime, particularly breath-focused relaxation, appeared to mitigate pre-sleep arousal, reduce difficulty falling asleep, and lessen the frequency of nocturnal awakenings. Over the course of the week, repeated application of these techniques established a stable psychological framework and behavioral rhythm, the benefits of which extended into the immediate preoperative period (37, 38). From a neuroanatomical perspective, the external ear is innervated by multiple sensory nerves, including the auricular branch of the vagus nerve, auriculotemporal branch of the trigeminal nerve, greater auricular nerve, lesser occipital nerve, and contributions from the facial and glossopharyngeal nerves. Stimulation of auricular regions may therefore influence autonomic and central nervous system activity through vagal and trigeminal afferent pathways. In this context, auricular acupressure may complement MBSR by providing peripheral neuromodulatory input, whereas mindfulness practice primarily targets cognitive-emotional arousal and attentional regulation. Preliminary neuroimaging studies have suggested that auricular stimulation may be associated with regionally specific brain responses. Romoli et al. provided pilot fMRI evidence suggesting point-related specificity in auricular acupuncture, and Yeh et al. reported dynamic brain activity changes following auricular point acupressure in patients with chemotherapy-induced neuropathy (39, 40). The observed reductions in salivary cortisol levels in the intervention group further support the hypothesis that both components helped regulate hypothalamic–pituitary–adrenal (HPA) axis function, alleviating stress-related disruptions in sleep (41–44).

Interestingly, while the intervention group exhibited significantly lower AIS scores three days before surgery compared to the control group, this difference was no longer significant on the day before surgery. Several factors may account for this attenuation. The imminent nature of surgery often triggers a sharp rise in procedural anxiety, including fears about anesthesia, surgical risk, and postoperative recovery. These acute psychological stressors may override the benefits of prior mindfulness or acupressure training. Furthermore, hospital admission itself introduces environmental and procedural disruptions—such as changes in routine, dietary restrictions, preoperative medical evaluations, and exposure to an unfamiliar setting—all of which can negatively impact sleep quality. In patients with spinal metastases, who frequently endure chronic pain and emotional volatility, such factors are likely to interact with underlying vulnerability, diminishing intervention efficacy. These findings highlight the need for future research to explore longer-duration interventions, booster sessions closer to surgery, or additional inpatient psychological support to better sustain improvements in sleep quality up to the point of operation.

In terms of pain management, the intervention group experienced significantly greater reductions in VAS scores compared to the control group. Mechanistically, mindfulness practice may enhance pain tolerance by encouraging individuals to adopt a non-judgmental, accepting attitude toward discomfort (29, 36). This perspective reduces catastrophizing and emotional reactivity, thereby disrupting the vicious cycle of “pain–anxiety–insomnia.” Repetitive engagement in breath-focused attention and body scan practices may also modulate pain perception by shifting cognitive and affective appraisal of nociceptive signals (45). In parallel, auricular acupressure—by stimulating sedative and analgesic points—may reduce sympathetic excitability, improve local microcirculation, and provide adjunctive pain relief. For patients with region-specific pain due to thoracic or lumbar metastases, tailored acupoint selection likely enhanced the specificity and effectiveness of the intervention (44, 46). Finally, the continuity of intervention delivery across outpatient, home-based, and inpatient settings, together with digital reminders and telephone follow-up, may have supported participant engagement during routine perioperative nursing practice. However, because structured adherence metrics were not uniformly archived, the relationship between intervention exposure and outcomes could not be quantitatively evaluated in this retrospective analysis.

## 5 Conclusion

Patients with spinal metastases frequently suffer from sleep disturbances and heightened anxiety during the preoperative period. These physical and psychological symptoms not only diminish quality of life but may also compromise postoperative recovery and perioperative safety. While pharmacological interventions remain the primary approach for managing tumor-related sleep disorders, long-term use carries risks including drug resistance, dependence, and impaired daytime functioning. In the context of personalized and holistic health care, there is growing interest in non-pharmacological strategies that are safe, non-invasive, and user-friendly. This study, grounded in a multidisciplinary collaborative framework, implemented a short-term preoperative intervention combining mindfulness-based stress reduction and auricular acupressure. The findings provide preliminary evidence supporting the feasibility and potential clinical value of this approach in improving objective sleep continuity indicators and stress-related outcomes among patients with spinal metastases. However, the effect on subjective insomnia symptoms measured by AIS should be interpreted cautiously because the association was attenuated after covariate adjustment.

## 6 Limitations

This study has several limitations. First, because group allocation was based on admission period rather than randomization, potential selection bias and temporal confounding cannot be fully excluded. Changes in patient case mix, clinical workflow, staffing, surgical scheduling, or perioperative management over time may have influenced the observed outcomes. Although the same inclusion and exclusion criteria were applied to both cohorts and both groups were treated in the same department by the same multidisciplinary perioperative care team, residual bias remains possible. Therefore, the findings should be interpreted as preliminary and require confirmation in prospective randomized controlled trials. In addition, detailed adherence metrics were not uniformly archived in a structured format because the intervention was implemented as part of routine perioperative nursing practice rather than a prospectively designed trial. Therefore, quantitative adherence indicators, such as session completion rate, cumulative practice duration, and response time to reminders, could not be reliably analyzed. Future prospective studies should predefine adherence indicators and automatically capture intervention exposure data. Second, this was a single-center study with a relatively small sample size, which may limit statistical power and generalizability. Although multivariable regression models were used to adjust for available demographic and clinical variables, residual clinical confounding remains possible. Detailed disease-related variables, such as tumor stage, tumor burden, anatomical severity of spinal metastasis, exact opioid dosage, and longitudinal changes in analgesic use, were not consistently available across retrospective records and therefore could not be reliably incorporated into the models. Finally, this study focused on preoperative outcomes and did not assess postoperative recovery, postoperative sleep outcomes, postoperative complications, or long-term follow-up effects of the intervention. Future prospective studies with larger samples and longer follow-up periods are needed to confirm these findings.

## Data Availability

The original contributions presented in the study are included in the article/**Supplementary Material**. Further inquiries can be directed to the corresponding authors.
