# Supplementary material for: A short-term mindfulness and auricular acupressure program for preoperative sleep fragmentation in patients with spinal metastases: a retrospective quasi-experimental study
Source: Front Psychiatry. 2026 Jul 24;17:1761276. doi: 10.3389/fpsyt.2026.1761276 (PMC13448551; doi:10.3389/fpsyt.2026.1761276)
Supplement: Supplementary file 1 [file Supplementaryfile1.docx]

**Supplementary Materials**

To ensure sufficient statistical power for detecting meaningful differences in the primary outcome, the SAS scores, a priori sample size estimation was performed. Based on the observed means and standard deviations of SAS scores from preliminary data (mean difference δ=3.92, pooled standard deviation σ=2.91), he following parameters were used: a two-sided significance level of 0.05 (*Z*_1-_*_α_*_/2_=1.96) and a desired power of 95% (*Z*_1-_*_β_*=1.96). The minimum required sample size per group was calculated using the formula for comparing two independent means:

$$n=\left( \frac{(Z_{1-\alpha/2}+Z_{1-\beta})\times\sigma}{\delta} \right)^{2}$$

Therefore, the minimum sample size required per group was approximately 8 participants. Given that the actual sample size in this study is 46 participants per group, it is well above the calculated minimum, ensuring adequate power to detect meaningful differences in anxiety levels between groups. Additionally, secondary outcomes such as SC, AIS, and WASO exhibited larger effect sizes, indicating that the current sample size is also sufficient to detect significant effects across all measured variables.
